# Anti-Tumour Effects of a Specific Anti-ADAM17 Antibody in an Ovarian Cancer Model *In Vivo*

**DOI:** 10.1371/journal.pone.0040597

**Published:** 2012-07-11

**Authors:** Frances M. Richards, Christopher J. Tape, Duncan I. Jodrell, Gillian Murphy

**Affiliations:** 1 Pharmacology & Drug Development Group, Cancer Research UK Cambridge Research Institute, and Department of Oncology, University of Cambridge, Cambridge, United Kingdom; 2 Proteases and Tumour Microenvironment Group, Cancer Research UK Cambridge Research Institute, and Department of Oncology, University of Cambridge, Cambridge, United Kingdom; Ludwig-Maximilians University, Germany

## Abstract

ADAM 17 (TNF-α converting enzyme, TACE) is a potential target for cancer therapy, but the small molecule inhibitors reported to date are not specific to this ADAM family member. This membrane-bound metalloproteinase is responsible for ectodomain shedding of pathologically significant substrates including TNF-α and EGFR ligands. The aim of this study was to evaluate the pharmacokinetics, pharmacodynamics and anti-tumour efficacy of the first specific inhibitor, an anti-human ADAM17 IgG antibody, clone D1(A12). We used intraperitoneal xenografts of the human ovarian cancer cell line IGROV1-Luc in Balb/c nude mice, chosen because it was previously reported that growth of these xenografts is inhibited by knock-down of TNF-α. *In vitro*, 200 nM D1(A12) inhibited shedding of ADAM17 substrates TNF-α, TNFR1-α, TGF-α, amphiregulin (AREG), HB-EGF and IL-6Rα, from IGROV1-Luc cells, (4.7 nM IC_50_ for TNF-α shedding). In IGROV1-Luc xenografts *in vivo*, D1(A12) IgG showed pharmacokinetic properties suitable for efficacy studies, with a single i.p. dose of 10 mg/kg D1(A12) sufficient to maintain IgG plasma and ascites fluid concentrations above 100 nM for more than 7 days. The plasma half life was 8.6 days. Next, an efficacy study was performed, dosing D1(A12) or anti-human TNF-α antibody infliximab at 10 mg/kg q7d, quantifying IGROV1-Luc tumour burden by bioluminescence. D1(A12) IgG showed a significant reduction in tumour growth (p = 0.005), 56% of vehicle control. Surprisingly, D1(A12) did not reduce the concentration of circulating human TNF-α, suggesting that another enzyme may compensate for inhibition of ADAM17 *in vivo* (but not *in vitro*). However, D1(A12) did show clear pharmacodynamic effects in the mice, with significant inhibition of shedding from tumour of ADAM17 substrates TNFR1-α, AREG, and TGF-α (4–15-fold reductions, p<0.0001 for all three). Thus, D1(A12) has anti-ADAM17 activity *in vivo*, inhibits shedding of EGFR ligands and has potential for use in EGF ligand-dependent tumours.

## Introduction

TNF-α converting enzyme (TACE, ADAM17) is a membrane-bound metalloproteinase responsible for cleavage and ectodomain shedding of TNF-α, EGFR ligands and other pathologically significant substrates. Dysregulation of ectodomain shedding is associated with autoimmune and cardiovascular diseases, neurodegeneration, infection, inflammation and cancer [1].

ADAM17 has been implicated in many cancers. It is overexpressed in ovarian cancer [2], breast cancer [3], [4], pancreatic ductal adenocarcinoma [5], colorectal carcinoma [6], gastric cancer stem cells [7], GIST [8], non-small cell lung carcinoma [9], and head and neck cancer [10], [11]. ADAM17 is reported to have a direct functional role in controlling proliferation and/or migration of cells derived from many tumour types [12], [13], [14], [15], [16], [17], [18], [19], [20], and it has been implicated in controlling endothelial cell migration [21] and pathological angiogenesis [22], [23], which is also relevant to tumour growth. ADAM17 can be activated by chemotherapy, resulting in growth factor shedding, hence contributing to resistance in colorectal cancer models [15], [24]. ADAM17 may also contribute to resistance to trastuzumab (Herceptin^TM^) in breast cancer [25]. Thus, ADAM17 makes an attractive target for development of inhibitors, which would have broad spectrum therapeutic potential in the treatment of patients with cancer.

Small molecular inhibitors of ADAM17 have been developed but none have yet shown complete specificity for ADAM17. For example, INCB3619 and INCB7839 inhibit ADAM10, ADAM17 and matrix metalloproteinases MMP2, MMP12 and MMP15 [9], [26], [27], [28]. The two ADAM17 inhibitors which have progressed the furthest in development [29] are DPC 333 (BMS-561392) [30] and TMI-005 (apratastat) [31]. These both show some inhibitory activity against other MMPS and did not progress beyond Phase II trial because of toxicity or lack of efficacy. Broad spectrum MMP inhibitors (marimastat, prinomastat, tanomastat), that also inhibit the ADAMs, have not progressed beyond phase III clinical trials due to lack of efficacy and significant toxicity [32], [33], [34], [35], suggesting that specificity is key when developing metalloproteinase inhibitors.

A specific human ADAM17 inhibitory antibody, D1(A12), has recently been developed, which inhibits the proteolysis of ADAM17 substrates (TNF-α, AREG, etc.,) in cancer cells *in vitro* [36]. The aim of the studies reported here was to assess the suitability of the D1(A12) antibody for therapeutic use, by investigating its pharmacokinetics, pharmacodynamics and anti-tumour efficacy in mice. We chose to use the IGROV1-Luc model of ovarian cancer, which has been reported to be TNF-α- dependent [37].

High levels of expression of TNF-α have been observed in ovarian carcinoma [38], [39], [40], particularly of the high grade serous type [41], and an anti-TNF-α antibody, infliximab (Remicade^TM^), has been investigated for its efficacy against ovarian cancer [42]. The IGROV1-Luc model is a human tumour xenograft model of intraperitoneal disseminated ovarian carcinoma. IGROV1-Luc cells secrete TNF-α and knockdown of TNF-α expression by transfection of IGROV1-Luc cells with anti-TNF-α shRNA was reported previously to inhibit tumour growth [37]. Inhibition of ADAM17 by D1(A12) antibody was expected to inhibit TNF-α secretion and thereby inhibit growth of IGROV1-Luc tumours *in vivo*. For comparison, a group of mice were treated with infliximab, which was expected to directly reduce functional human TNF-α, also leading to inhibition of growth of the IGROV1-Luc tumours.

## Materials and Methods

### Antibodies and chemicals

Isolation and purification of human anti-ADAM17 antibody D1(A12) has been described previously [36]. Briefly, D1(A12) IgG was expressed by transfection of HEK293 cells and conditioned medium was collected. The antibody was purified from the medium by chromatography on 2 protein L columns (Pierce) followed by 2 Melon Gel columns (Pierce) and AKTA FPLC, and then dialysed into sterile Phosphate Buffered Saline (PBS), pH 7.4 and filter-sterilised. Infliximab (Remicade^TM^, Janssen Biotech Inc.), a kind gift from Prof. Peter Taylor (Kennedy Institute of Rheumatology), was dissolved in sterile saline. Control human plasma IgG (R&D Systems 1-001-A) was used as a control in some cell-based assays. N-TIMP-3 was prepared as described by Lee *et* *al* [43]. Phorbol-12-myristate-13-acetate (PMA) was purchased from Sigma, dissolved in DMSO to 25 μg/ml and diluted to 100 ng/ml in culture medium for assays.

### Cell culture

The IGROV1 human ovarian cancer cell line [44] was infected with a lentiviral vector containing a luciferase reporter construct [37], and this IGROV1-Luc cell line was a kind gift from Professors Fran Balkwill and Ian McNeish. The cells were grown in RPMI-1640 medium containing 10% Fetal Bovine Serum. The cell line tested negative for mycoplasma and the STR genotype corresponded to that reported for IGROV1 cells in the NCI60 panel. Also, the reported p53 mutation (Y126C) was confirmed in these cells by DNA sequencing.

### Animal Studies

All mouse studies were performed in accordance with the UK Animals (Scientific Procedures) Act 1986, under project licence number 80/2346, with approval from the CRUK Cambridge Research Institute Animal Ethics Committee and following current UK Guidelines [45]. Balb/c nude female mice were obtained from Charles River Laboratories and were used at 6–10 weeks old (8 – 10 weeks for the PK study and 6 – 8 weeks for the efficacy study). Mice were housed in groups in sterile Individually Ventilated Cages. Mice were injected i.p. with 5×10^6^ IGROV1-Luc cells and were observed daily for tumour growth and clinical signs. Tumour burden was quantified weekly by bioluminescent imaging, as described below.

Both antibodies were diluted in sterile PBS, pH 7.4 for dosing into mice at 10 mg/kg. For the PK study at least 2 mice per time point were dosed i.p. with 10 mg/kg in a volume of 9.5 ml/kg. The tumour-bearing mice were dosed for PK studies either 34 days after tumour implantation (for shorter time points), or 27 days after implantation for 7 – 9 day time points. The concentrations of D1(A12) and infliximab in plasma, ascites fluid and tumour homogenates were measured by quantitative ELISA as described below. For the efficacy study the mice were randomised into 3 groups and dosed i.v. with the antibodies at 10 mg/kg in a dose volume of 4.76 ml/kg, or with sterile PBS as a vehicle control. The first dose was given on day 4 after tumour cell injection, immediately after the first bioluminescent imaging session, and every 7 days thereafter until day 32. On day 32 the mice were imaged for the last time and given a final dose, then they were killed the following day, day 33, 20 hours after the last dose.

Blood was collected in lithium heparin tubes, centrifuged at 18,800 g for 5 minutes and plasma collected. Ascites fluid was collected from the abdominal cavity and centrifuged at 18,800 g for 5 minutes to remove any cells. Plasma and ascites fluid were then snap-frozen and stored at −80°C. Organs and tumour were collected post-mortem with part snap-frozen and part fixed in Neutral Buffered Formalin (Sigma). Formalin-fixed tissues were paraffin embedded, sectioned and stained with haematoxylin and eosin. For analysis of tumour tissue by ELISA, weighed pieces of frozen tissue (approximately 20 mg) were homogenised in MK-28R tubes containing metal beads using a Precellys 24 homogeniser (Bertin Technologies, supplied by Stretton Scientific, UK) at 6000 rpm for 50 seconds, twice. The samples were homogenised at a concentration of 50 mg of tissue per ml of buffer (PBS, pH 7.4 with added protease inhibitor cocktail (Sigma) and phosphatase inhibitor cocktail 2 (Sigma) each diluted 1 in 100). The homogenates were centrifuged at 10,000 g for 10 minutes at 4°C and the supernatant was used for ELISAs (see below).

### Bioluminescent Imaging

Mice were anaesthetised using isoflurane then injected i.p. with 150 μg/g body weight D-Luciferin in PBS (Caliper Life Sciences) and bioluminescent imaging with a charge-couple device camera (IVIS 200, Xenogen, Alameda CA) was initiated 10 min after injection. Images were obtained for groups of 3 mice with a 13 cm field of view, binning (resolution factor) of 8 (medium), F stop  = 1, exposure  = 1 to 10 seconds.

Data were analysed using Living Image 3.2 Software (Xenogen) and presented as Average Radiance (units: photons/sec/cm^2^/steradian) for each mouse, from a constant sized Region of Interest drawn over the mouse abdomen. The mean and standard deviation were expressed for each treatment group. Significance between treated and vehicle groups was calculated using a t Test.

### Quantitative ELISA

For both D1(A12) and infliximab human IgGs, ELISA plates were coated with 50 μl/well 100 nM anti-human IgG (Abcam ab700) in PBS. After washing with PBS-Tween (0.1% v/v) and blocking with PBS-Milk (5% w/v), 50 μl of sample was added to each well (either 50 μl of plasma, tissue homogenate, or known concentrations of D1(A12) or infliximab IgG for the standard curves (0 – 1000 nM range, diluted in blank matrix (plasma or tumour homogenate from untreated mice)). After incubation and washing the human IgG was detected by incubating with anti-human-kappa light chain-HRP antibody (Bethyl A80-115P) (1∶2000 in PBS-milk), followed, after washing, by 50 μl of 3,3′,5,5′-tetremethylbenzidine (TMB) incubated at room temperature for 2 mins then quenched with 50 μl 1M HCl. Absorbance was measured at 450 nm. Pharmacokinetic parameters were calculated using a non-compartmental model in WinNonlin v5.1 (Pharsight).

ELISAs for ADAM17 substrates were performed using R&D Systems Duoset kits: human TNF-α (TNFSF1A, cat. No. DY210), human soluble TNFR1-α (TNFRSF1A, cat. No. DY225), human TGF-α (cat. No. DY239), human AREG (cat. No. DY262), human IL-6Rα (cat. No. DY227) and human HB-EGF (DY259). The DY210 kit was confirmed to be specific for human TNF-α by testing recombinant mouse TNF-α with this kit, there was no cross-reactivity.

Where tumour homogenates were used, raw nM concentrations in homogenate solution were converted to nmoles per mg tissue, and then using the assumption that tissue density is 1 g/ml, the molar concentration in tissue was calculated.

### Immunohistochemistry

Immunohistochemistry was performed on formalin-fixed, paraffin-embedded sections of tumour and liver after antigen retrieval by enzyme digestion (proteinase k) at 37°C for 10 minutes. The human IgG with which the mice had been dosed (D1(A12) or infliximab) was detected by binding of a Biotin-SP-conjugated AffiniPure F(ab')2 fragment donkey anti-human IgG (H+L) (Jackson ImmunoResearch Laboratories, Inc.), followed by staining with DAB+ chromogen. Slides were scanned using a ScanScopeXT (Aperio Technologies, Inc.) at 20X magnification and scanned images were viewed using Spectrum v10 software (Aperio Technologies Inc). No image manipulation was performed.

## Results

### Effect of D1(A12) antibody in IGROV1-Luc cells *in vitro*

**Figure 1:**
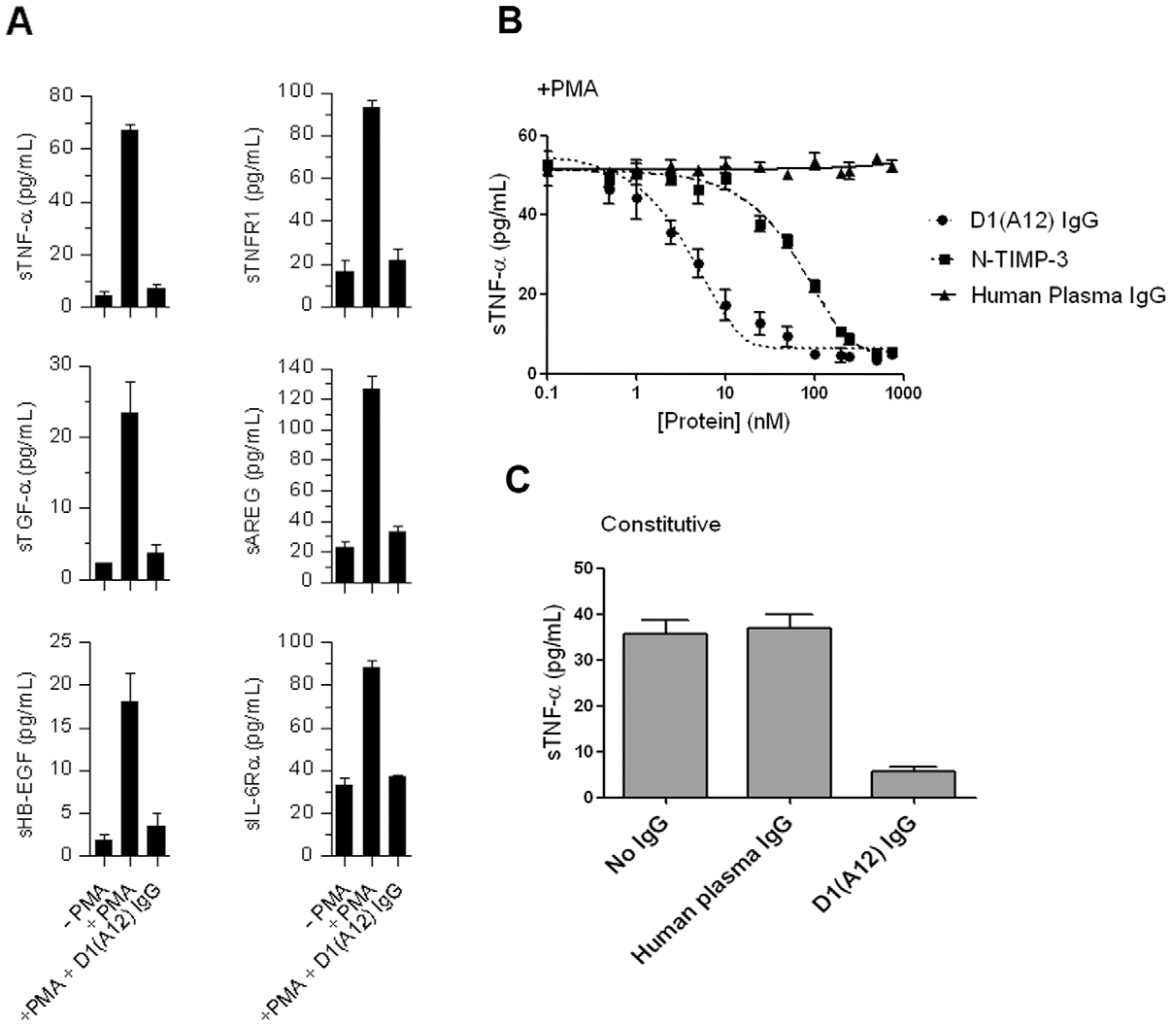
*In vitro* activity of D1(A12) antibody. (A) D1(A12) IgG inhibits PMA-induced shedding of ADAM17 substrates into IGROV1-Luc cell culture medium. Medium was collected 90 minutes after addition of PMA (100 ng/ml), D1(A12) IgG (200 nM) or solvent control. The proteins were quantified by ELISA. (B) Dose-dependent inhibition of TNF- α shedding by 1 hour pretreatment with D1(A12), N-TIMP-3 or control human plasma IgG prior to PMA stimulation. (C) D1(A12) IgG inhibits constitutive shedding of TNF-α from IGROV1-Luc cells into culture medium. Medium was collected after 48 hours of incubation with or without IgGs at 200 nM. Error bars show the standard deviation.

IGROV1-Luc tumour cells *in vitro* secreted TNF-α, TNFR1-α, TGF-α, AREG, HB-EGF and IL-6Rα, all known substrates for the shedding activity of ADAM17, into the culture medium even when unstimulated (Figure 1A). When stimulated with PMA the concentrations of these proteins shed into the medium increased at least 5-fold (apart from IL-6R α, 2.7-fold increase). This PMA-stimulated increase was inhibited by the addition of 200 nM D1(A12) IgG (Figure 1A). The inhibition of TNF-α shedding by D1(A12) was dose-dependent, with an IC_50_ in IGROV cells of 4.7 nM (Figure 1B). D1(A12) was a more potent inhibitor of TNF-α shedding than N-TIMP-3 (IC_50_ of 72 nM), a natural metalloproteinase inhibitor previously shown to inhibit ADAM17 [43]. D1(A12) IgG also inhibited constitutive shedding of TNF-α into the medium over a longer period (Figure 1C). D1(A12) did not inhibit proliferation of IGROV1-Luc cells *in vitro* in the presence of normal growth medium (data not shown), which is consistent with the effect of the TNF-α shRNA on IGROV1-Luc cells as previously reported [37].

### IGROV1-Luc Tumour growth *in vivo*

**Figure 2:**
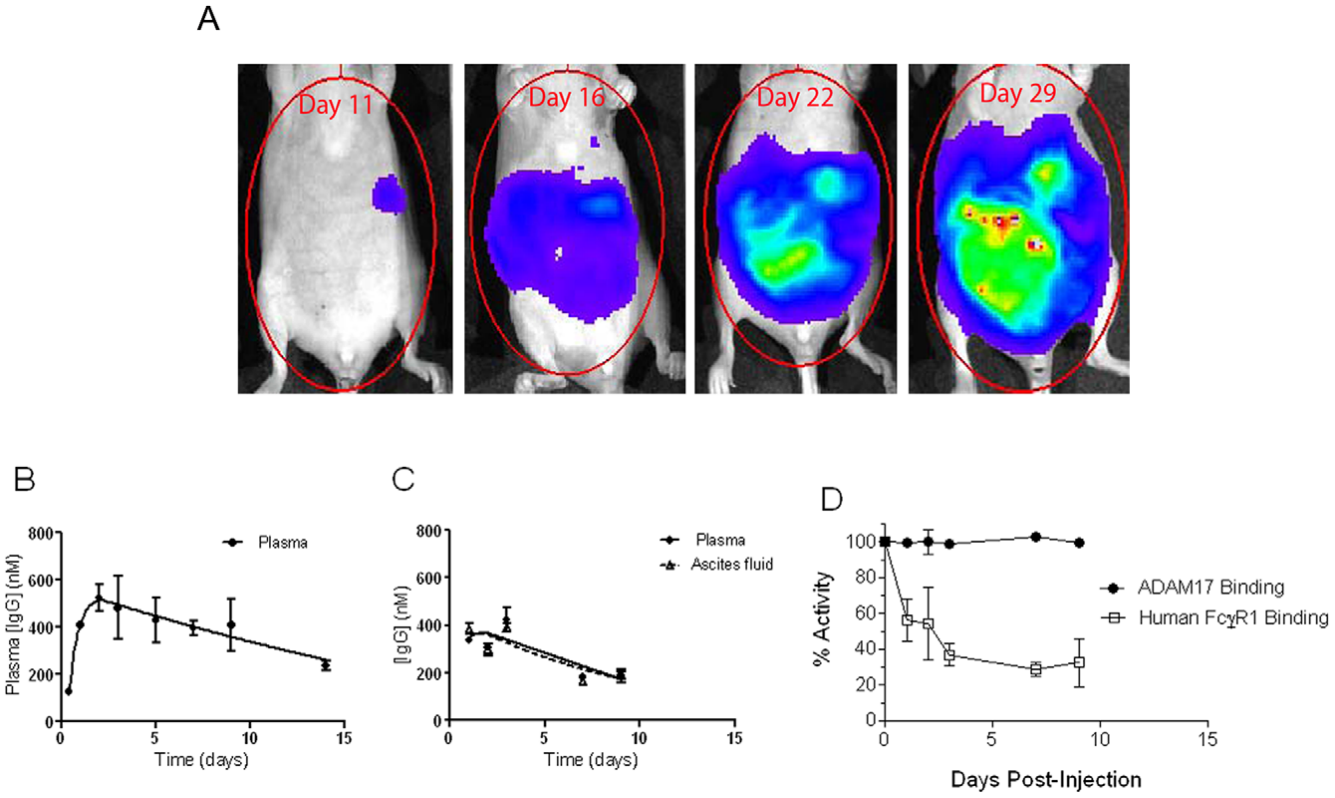
The IGROV1-Luc xenograft model and D1(A12) pharmacokinetics. (A) Example of the growth of IGROV1-Luc intraperitoneal tumour, as measured by bioluminescence, from 11 to 29 days after injection of the cells. (B and C) Pharmacokinetics of D1(A12) in nude mice after a single dose of 10 mg/kg i.p. N = 2 or more mice per time point. Error bars represent the standard error of the mean (B) Plasma concentrations in non-tumour-bearing mice. (C) Plasma and ascites fluid concentrations in IGROV1-Luc tumour-bearing mice. (D) D1(A12) IgG in mouse plasma: capacity for binding to ADAM17 and FcγR1. Plasma was collected at the indicated times after dosing the mice with a single dose of 10 mg/kg D1(A12). Binding activity *in vitro* was compared to the binding capacity of D1A12 stock solution. Error bars represent the standard error of the mean.

The IGROV1-Luc cells grew and disseminated intraperitoneally in nude mice (Figure 2A), as previously reported [37], with individual tumour masses showing a histological appearance consistent with human high grade serous ovarian adenocarcinoma (M. Jimenez-Linan, personal communication). The largest deposits of tumour were in the pancreas, omentum and mesentery of all mice. By the endpoint around day 32 – 35, the tumour-bearing mice had developed peritoneal exudate (ascites fluid). Human TNF-α, TNFR1-α, TGF-α, amphiregulin (AREG), and HB-EGF (ADAM17 substrates) were detected in the circulation of IGROV1-Luc tumour-bearing mice, with high concentrations in the ascites fluid and lower concentrations in the plasma (as shown in the vehicle control groups of the efficacy study, shown later).

### Pharmacokinetics of D1(A12) IgG

The pharmacokinetics (PK) of D1(A12) antibody were investigated using a single 10 mg/kg dose i.p., in non-tumour-bearing mice (Figure 2B). PK parameters were calculated for non-tumour-bearing mice using the WinNonLin noncompartmental analysis programme: plasma C_max_ = 523+/−58 nM, T_max_ 2 days, half life 8.6 days. More limited sampling was then performed in mice bearing IGROV1-Luc tumours (Figure 2C), in which the D1(A12) IgG showed similar kinetics to the non-tumour bearing mice. After a 10 mg/kg dose i.p. the C_max_ was 425+/−51 nM in plasma and 391+/−19 nM in ascites fluid, lower than the plasma C_max_ in the mice without tumours. These data were sufficient to predict that circulating D1(A12) concentrations of above 100 nM can be maintained by dosing 10 mg/kg once every 7 days. 100 nM D1(A12) is sufficient concentration to cause maximal inhibition of ADAM17 function in IGROV1 cell culture (Figure 1B).

The detection of D1(A12) antibody by ELISA does not necessarily mean that the antibody had retained its activity, as it could be partially denatured, so the binding activity of the plasma D1(A12) antibody was assessed (Figure 2D and Figure S1). The D1(A12) antibody from the plasma of mice at all time points from 1 to 9 days retained the ability to bind ADAM17 (100% at day 9, compared to the D1(A12) stock solution). In contrast, the ability of D1(A12) to bind to human FcγR1 decreased with time, with binding after 9 days in the mouse only 32% of the binding of D1(A12) stock solution.

### Efficacy studies and pharmacodynamics

**Figure 3:**
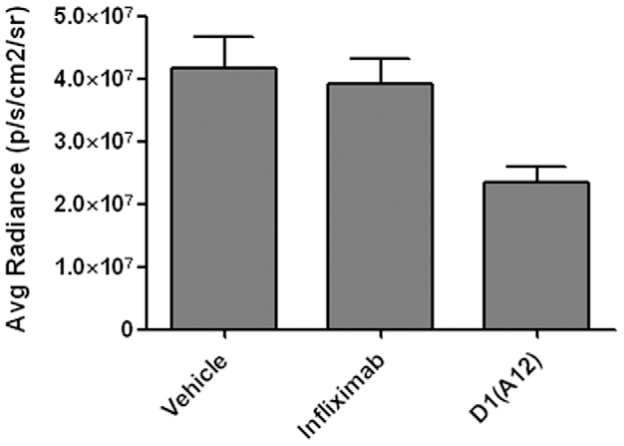
Inhibition of IGROV1-Luc tumour growth *in vivo* by D1(A12) IgG. Growth of IGROV1-Luc i.p. xenografts in mice dosed weekly with vehicle, 10 mg/kg infliximab or 10 mg/kg D1(A12), measured by bioluminescence. The mean and standard deviation of day 32 tumour burden is shown, expressed as Avg Radiance. N = 12 for vehicle, n = 8 for infliximab and N = 11 for D1(A12).

Having established that the D1(A12) antibody has suitable PK characteristics, we tested the effect of weekly dosing in IGROV1-Luc xenografts with 10 mg/kg D1(A12) (n = 11), in comparison with 10 mg/kg infliximab (n = 8) and PBS vehicle (n = 12). Prior to the first dose on day 4 after cell injection there was no significant difference in the tumour burden between the groups: Avg Radiance (x 10^6^ p/s/cm^2^/Sr) 2.71+/−1.35, 2.92+/−1.23 and 2.65+/−0.91 for vehicle, infliximab and D1(A12) groups, respectively. Tumour size at the endpoint on day 32 is presented in Figure 3. The D1(A12) group had a significantly smaller tumour burden (Avg Radiance x 10^6^ p/s/cm^2^/Sr) on day 32 of 23.3+/−9.1 compared to the vehicle group (41.8+/−17.2, p = 0.005). The mean tumour burden in the D1(A12) group at the endpoint was 56% of the vehicle control. In contrast there was no inhibition of tumour growth in mice treated with infliximab compared to vehicle, with the tumour burden of 39.1+/−11.6 in the infliximab group (p = 0.68).

**Figure 4:**
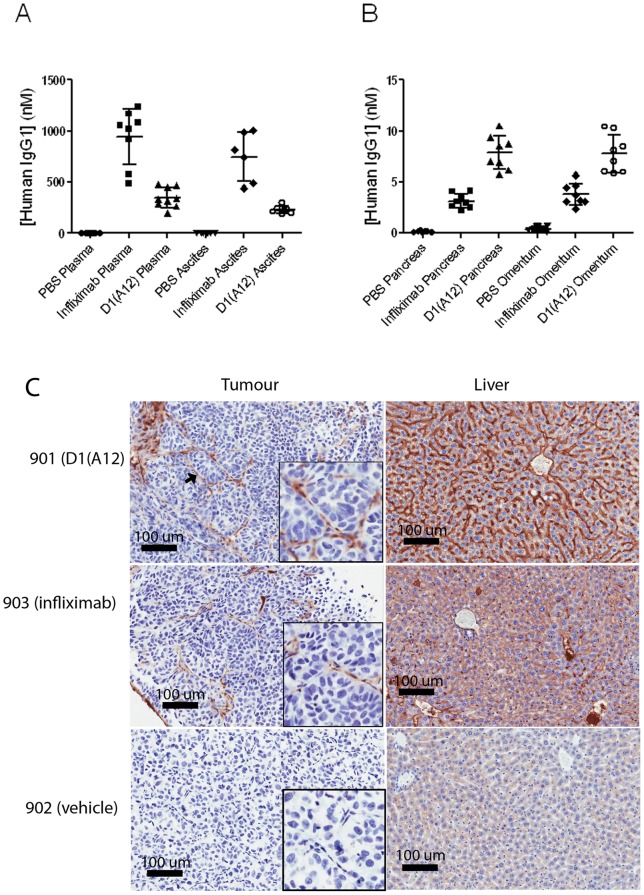
Distribution of D1(A12) and infliximab IgG *in vivo* at the end of the efficacy study. Samples were taken at endpoint (day 33), 20 hours after the final antibody dose. (A) Concentrations in plasma and ascites fluid. Horizontal bars represent the mean and standard deviation. (B) Concentrations in homogenates of tissue from tumour in pancreas and omentum. (C) Immunohistochemistry to detect human IgG in IGROV1-Luc tumour and liver. Top: mouse ID 901, treated with D1(A12), with an arrow marking the position of a blood vessel; middle: mouse ID 903, treated with infliximab; bottom: mouse ID 902, treated with vehicle. Images were taken at 20X magnification.

To confirm that therapeutic concentrations of antibody had been achieved, the concentration of D1(A12) and infliximab was determined in the plasma and ascites fluid (Figure 4A) and in tumour homogenates (Figure 4B). As expected, no human IgG was detected in any of the samples from the vehicle-treated group. D1(A12) and infliximab were present at high concentrations in plasma and ascites fluid in the appropriate treatment group, and both antibodies were detectable in tumour from both pancreas and omentum. The concentration of D1(A12) was higher in tumour than infliximab, but infliximab was higher in plasma and ascites fluid than D1(A12).

To investigate the distribution of D1(A12) and infliximab in the tumour tissue, anti-human IgG immunohistochemistry was performed on paraffin sections from the tumours (Figure 4C). The two therapeutic antibodies were detectable as strong IHC staining in the liver, and both were also detectable in the tumour tissue, but the distribution appeared to be confined to the blood vessels.

**Figure 5:**
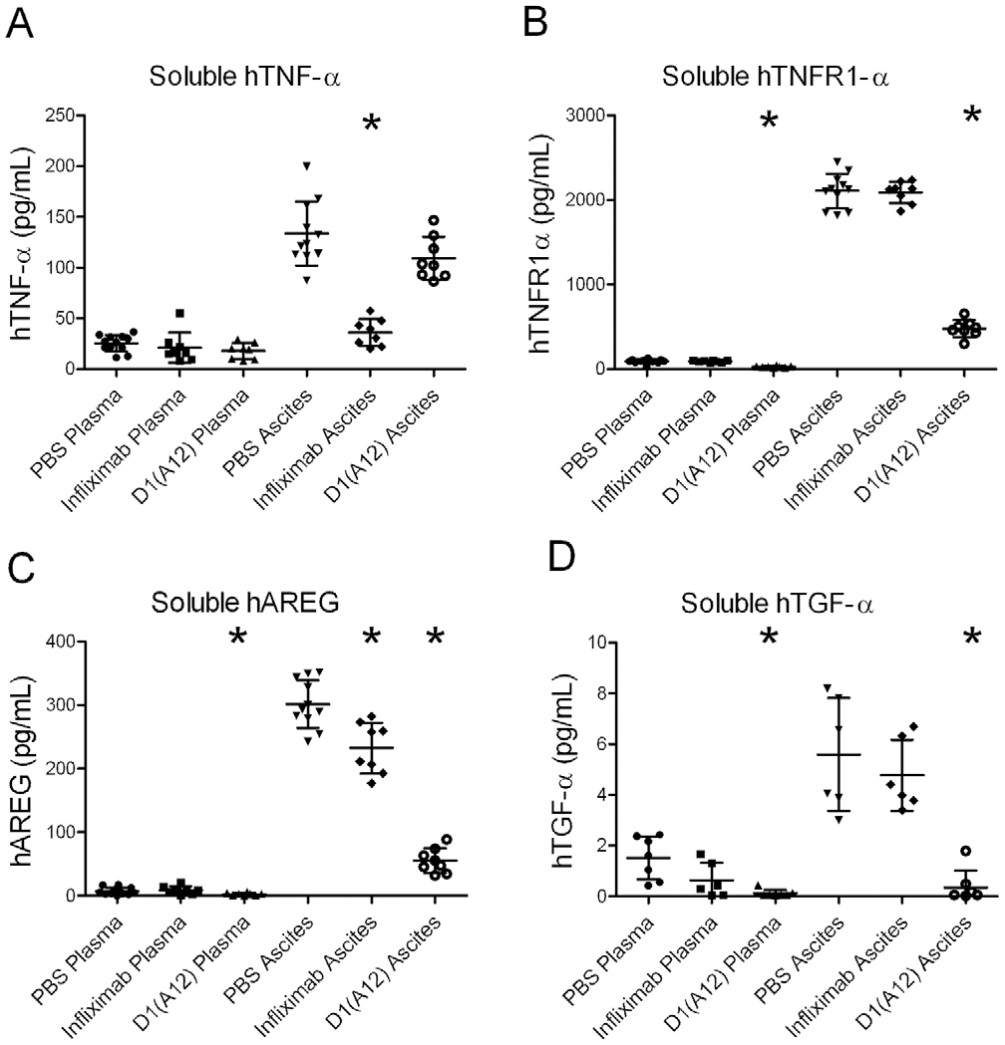
Inhibition of shedding of ADAM17 substrates by D1(A12) and infiximab. Concentrations of the cleaved products of ADAM17 substrates in plasma and ascites fluid at the endpoint (day 33), as measured by ELISA. Horizontal bars represent the mean and standard deviation. Asterisks show the groups significantly different (P<0.05) from the PBS control group in the same fluid type.

To determine whether either antibody had pharmacodynamic effects we analysed the concentrations in plasma and ascites fluid of the products of ADAM17 sheddase activity, i.e. soluble human TNF-α, soluble TNFR1-α, TGF-α and amphiregulin (AREG) (Figure 5). All four of the proteins analysed showed significantly higher concentrations in ascites fluid than in plasma. As expected, there was a significant reduction in sTNF-α in the ascites fluid of infliximab-treated mice when compared to vehicle (3.7-fold reduction, p<0.0001). Surprisingly, there was no significant reduction in sTNF-α in the D1(A12)-treated mice (P = 0.06). However, D1(A12) did show clear pharmacodynamic effects by significantly reducing the ascites fluid concentrations of soluble TNFR1-α (4.4-fold reduction, P<0.0001), AREG (5.4-fold reduction, P<0.0001) and TGF-α (15-fold reduction, P<0.0001). D1(A12) also reduced significantly the plasma concentrations of soluble TNFR1-α (P<0.0001), AREG (P = 0.012) and TGF-α (P = 0.005).

## Discussion

We have investigated the biological activity of D1(A12), a monoclonal antibody specific for human ADAM17, which inhibits ADAM17 function by cross-domain inhibition of the ectodomain. We have demonstrated that the antibody (at nanomolar concentrations) inhibits shedding of ADAM17 substrates including TNF-α and AREG in IGROV1-Luc cells *in vitro*.

The PK properties of the D1(A12) IgG in mice were found to be suitable for efficacy studies. The plasma half life is consistent with published values for the half life of human IgG antibodies in mouse plasma [46]. The plasma and ascites C_max_ were lower in the tumour bearing mice than the plasma C_max_ in the mice without tumours. However, this is not surprising given the larger total body fluid volume in the tumour-bearing mice due to the ascites fluid compartment. The plasma and ascites fluid concentrations of D1(A12) IgG were very similar within each individual mouse, from both the PK and efficacy studies, suggesting that the antibody is able to equilibrate between these two compartments after either IP or IV dosing. We have demonstrated that D1(A12) antibody is structurally stable in the mouse circulation, retaining the ability to bind to human ADAM17 up to 9 days after dosing. However, the ability of D1(A12) to bind to human FcγR1 decreased with time in the mouse plasma, such that only 32% of the binding activity remained after 9 days in the mouse. The reduced FcγR1 binding capacity may be due to soluble Fc-receptors present in mouse serum. For example, mouse FcRn binds human IgG1 with high affinity [47] and is responsible for the long *in vivo* half-life of antibodies [48].

The PK properties and stability of D1(A12) IgG in the mouse circulation indicated that weekly dosing should maintain concentrations high enough to be biologically active *in vivo*, so efficacy studies were performed in the IGROV1-Luc model. D1(A12) antibody showed anti-tumour effects, with the tumour burden at the end of the study approximately 56% of the vehicle control group. This was a modest anti-tumour effect, and some possible explanations for this are discussed below.

Tumour homogenates had concentrations of D1(A12) IgG above the cellular IC_50_, but analysis by IHC showed limited penetration beyond the tumour blood vessels. Similar limited penetration has been observed in xenograft tumours for the high affinity anti-HER2 antibody trastuzumab [49], which is nevertheless an effective drug *in vivo*. Distribution of monoclonal antibodies into tumour tissue has been shown to be limited by penetration through capillary walls and also by additional factors once the antibody has crossed the blood vessel wall [50]. Penetration of D1(A12) may be better in tumours with more permeable vessels than those in the IGROV1 tumours. However, limited tumour penetration of D1(A12) IgG in the IGROV1-Luc tumours did not prevent the antibody causing clear pharmacodynamic effects (shedding of ADAM17 substrates, see below). Interestingly, D1(A12) concentration was higher in tumour than infliximab, but lower in plasma and ascites fluid than infliximab, perhaps reflecting the location of the target for each antibody (ADAM17 at the tumour cell plasma membrane, versus circulating TNF-α which is present at high concentrations in the plasma and ascites).

To determine whether the antibodies were hitting their targets and having pharmacodynamic effects, ELISA assays of ADAM17 substrates were performed on plasma and ascites fluid samples at the end of the efficacy study. Vehicle-treated mice showed much higher concentrations of soluble TNF-α, TNFR1-α, AREG and TGF-α in ascites fluid than in plasma, suggesting that these proteins do not freely equilibrate between the plasma and ascites compartments, in contrast to the dosed IgGs which did equilibrate between the compartments.

Comparison of the D1(A12) treated group with the vehicle controls showed that D1(A12) did significantly inhibit shedding of ADAM17 substrates, the EGFR ligands TGF-α and AREG, and TNFR1-α. However, D1(A12) did not significantly inhibit TNF-α shedding *in vivo*, which was surprising because TNF-α shedding was inhibited by D1(A12) in the IGROV1-Luc cells *in vitro*. The lack of inhibition of TNF-α shedding *in vivo* might explain the relatively modest antitumour effect of the antibody, because TNF-α was thought to be a key driver of tumour growth in this model, based on the shRNA work of Kulbe, *et* *al* [37], but the infliximab data confounds this hypothesis (see below).

ADAM17 has been considered the key enzyme responsible for shedding of TNF-α, but our results with the D1(A12) antibody suggest that another enzyme may compensate when ADAM17 activity is inhibited in IGROV1-Luc cells grown *in vivo* (but not *in vitro*). This may be ADAM10: ADAM10 has shown TNF-α sheddase activity in ADAM17-deficient murine fibroblasts [51] and has been implicated in TNF-α production in mantle cell lymphoma [52]. Also, Hikita et al [53] showed that while ADAM17 is indeed the major TNF-α sheddase in macrophages, ADAM10 is also a TNF-α sheddase in adenovirus-transformed human embryo kidney 293A cells, NIH3T3 mouse embryo fibroblasts and murine endothelial cells. Also, when overexpressed, ADAM19 may be capable of contributing to TNF-α shedding [54]. These data suggest that for inhibition of TNF-α shedding from tumours *in vivo*, inhibition of both ADAM17, ADAM10 and ADAM19 may be required. In the *in vivo* studies the tumour cells would have been exposed to D1(A12) for 28 days, and if the compensatory mechanisms were slow to develop, this could explain why this compensation was not observed in the *in vitro* assays which were performed over 48 hours or less. Another possible explanation for the lack of inhibition of TNF-α shedding *in vivo* is that shedding in trans could be occurring, where murine ADAM17 on host cells, which would not be regulated by D1(A12), could cleave TNF-α on adjacent human tumour cells. To our knowledge trans-shedding has not been reported for ADAM17, but it has for ADAM10 cleavage of ephrin A5 [55]. This would suggest that TNF-α shedding was confined to highly active regions of host-tumour interaction such as at the invading margins. Whatever the mechanism for the sustained shedding of TNF-α *in vivo*, it is striking that shedding of the other ADAM17 cleavage products were inhibited by D1(A12). It is possible that the difference relates to the type of transmembrane proteins involved: pro-TNF-α is a Type II transmembrane protein, whose C-terminus is extracellular, whereas pro-AREG and the other EGFR ligands are Type I transmembrane proteins, whose N-termini are extracellular. This might confer a difference in specificity for other metalloproteinases in certain contexts, or the ability to be a substrate for shedding in trans. Another possible explanation for the difference between the ADAM17 substrates is that TNF-α shedding might occur deeper within the tumour mass (where antibody penetration may be limiting) than the shedding of the EGFR ligands. If the D1(A12) was unable to inhibit TNF-α shedding by ADAM17 deep in the tumour this pharmacokinetic feature could also explain the discrepancy between the *in vitro* and *in vivo* studies, as the cells *in vitro* would all be exposed to the antibody.

We used the specific human TNF-α antibody infliximab in our efficacy studies as a positive control, expecting it to inhibit growth of IGROV1-Luc tumours. TNF-α shedding was significantly inhibited in the infliximab group compared to the vehicle, but surprisingly infliximab did not inhibit tumour growth, in contrast to the published study showing that TNF-α shRNA knockdown did inhibit IGROV1-Luc tumour growth *in vivo* [37]. What could be the explanation for the difference between antibody and shRNA? With the shRNA the TNF-α was knocked down before the cells were implanted into the mice, whereas with our antibody efficacy study the TNF-α was knocked down only after the tumour had established in the mice, 4 days after implantation, so it is possible that TNF-α is required for the establishment of the tumour but not thereafter. However, we have pre-treated cells with infliximab prior to implantation and started dosing with infliximab at the time of implantation and this still did not inhibit tumour initiation or subsequent growth (data not shown). Another possibility is that endogenous mouse TNF-α was able to compensate for reduced human TNF-α in the infliximab-treated mouse model, because it is known that mouse TNF-α can bind human TNFR1 and TNFR2 [56]. However, Salako, *et* *al*., have recently confirmed that infliximab did not reduce tumour growth in this IGROV1 model, even when co-administered with an anti-mouse TNF-α antibody [57]. Thus, signalling by murine TNF-α is unlikely to explain the difference in antitumour activity between knockdown of TNF-α protein levels and the knockdown by shRNA. It is possible that other factors could compensate for the infliximab-induced reduction in TNF-α, but we would expect that these compensating factors would also have counteracted the effect of TNF-α knockdown, and that was not observed in the studies by Kulbe, et al [37]. As well as paracrine signalling by soluble TNF-α, juxtacrine signalling by transmembrane proTNF-α has been reported in a variety of cell types [58], [59], [60]. However, infliximab can effectively inhibit both soluble and transmembrane TNF-α [61] and should therefore inhibit juxtacrine signalling also. It is possible that the limited penetration of infliximab into the tumour tissue resulted in active transmembrane TNF-α remaining deep in the tissue, which continued to signal in a juxtacrine manner and supported tumour growth, even when infliximab had reduced the concentration of circulating sTNF-α. This could explain the discrepancy between the infliximab and siRNA results and between the *in vitro* and *in vivo* results.

While inhibition of ADAM17 by D1(A12) did not significantly inhibit TNF-α shedding it did efficiently inhibit shedding of other ADAM17 substrates, the EGFR ligands TGF-α and AREG, suggesting that other ADAMs do not compensate for loss of ADAM17 activity on these proteins *in vivo*. Thus, the D1(A12) specific ADAM17 inhibitor which showed modest antitumour effects in the IGROV1-Luc model may show more effective antitumour activity in tumours that are dependent on EGFR ligand signalling.

## Supporting Information

Figure S1 **Analysis of the binding capacity of the D1(A12) IgG in mouse plasma.** Each line represents the plasma from one mouse, taken from the tumour-bearing mice used in the PK study (ID number 809 to 820, with the timepoint in days shown in parentheses). Using the D1(A12) plasma IgG concentrations as determined in Figure 2C, the IgG was diluted to different concentrations and tested for binding by ELISA. (A) Binding to a control anti-human IgG, to confirm the dilutions of plasma D1(A12) are correct. (B) Binding to ADAM17. (C) Binding to human FcγR1.
(TIF)
