# Supplementary figures and images for: Anti-Tumour Effects of a Specific Anti-ADAM17 Antibody in an Ovarian Cancer Model In Vivo
Source: PLoS One. 2012 Jul 11;7(7):e40597. doi: 10.1371/journal.pone.0040597 (PMC3394719; doi:10.1371/journal.pone.0040597)

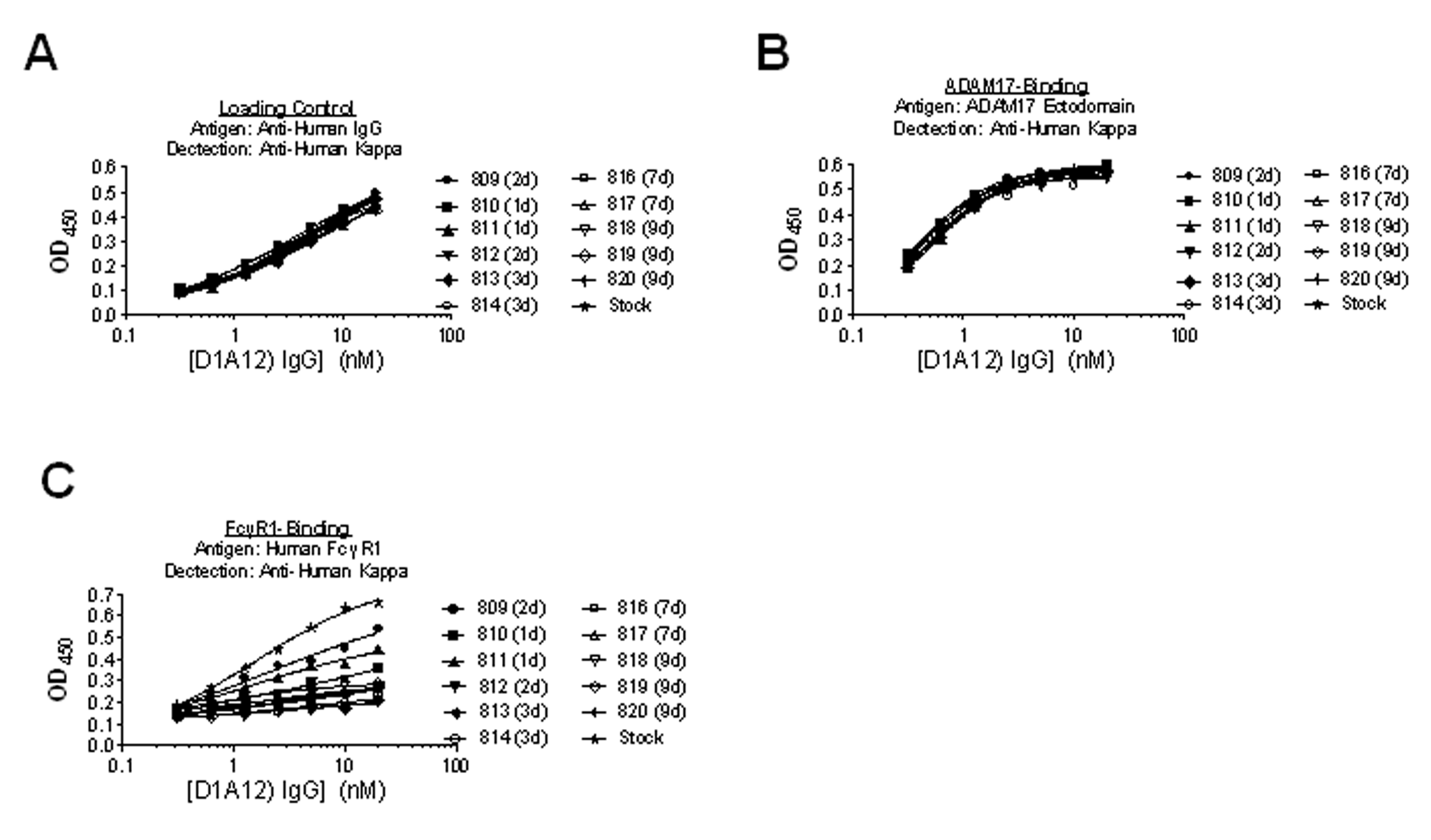

Supplement: Figure S1 — Analysis of the binding capacity of the D1(A12) IgG in mouse plasma. Each line represents the plasma from one mouse, taken from the tumour-bearing mice used in the PK study (ID number 809 to 820, with the timepoint in days shown in parentheses). Using the D1(A12) plasma IgG concentrations as determined in Figure 2C, the IgG was diluted to different concentrations and tested for binding by ELISA. (A) Binding to a control anti-human IgG, to confirm the dilutions of plasma D1(A12) are correct. (B) Binding to ADAM17. (C) Binding to human FcγR1. (TIF) [file pone.0040597.s001.tif]
